# Label-Free Sensing of Adenosine Based on Force Variations Induced by Molecular Recognition

**DOI:** 10.3390/bios5010085

**Published:** 2015-03-19

**Authors:** Jingfeng Li, Qing Li, Lucio Colombi Ciacchi, Gang Wei

**Affiliations:** 1 Hybrid Materials Interfaces Group, Faculty of Production Engineering, University of Bremen, Am Fallturm 1, 28359 Bremen, Germany; E-Mails: jinfeng@uni-bremen.de (J.L.); qing_li@uni-bremen.de (Q.L.); colombi@hmi.uni-bremen.de (L.C.C.); 2 Center for Environmental Research and Sustainable Technology (UFT), University of Bremen, Leobener Str., 28359 Bremen, Germany

**Keywords:** label-free sensing, adenosine, AFM, single-molecule force spectroscopy, aptamer, molecular recognition

## Abstract

We demonstrate a simple force-based label-free strategy for the highly sensitive sensing of adenosine. An adenosine ssDNA aptamer was bound onto an atomic force microscopy (AFM) probe by covalent modification, and the molecular-interface adsorption force between the aptamer and a flat graphite surface was measured by single-molecule force spectroscopy (SMFS). In the presence of adenosine, the molecular recognition between adenosine and the aptamer resulted in the formation of a folded, hairpin-like DNA structure and hence caused a variation of the adsorption force at the graphite/water interface. The sensitive force response to molecular recognition provided an adenosine detection limit in the range of 0.1 to 1 nM. The addition of guanosine, cytidine, and uridine had no significant interference with the sensing of adenosine, indicating a strong selectivity of this sensor architecture. In addition, operational parameters that may affect the sensor, such as loading rate and solution ionic strength, were investigated.

## 1. Introduction

Adenosine is a nucleoside composed of adenine and d-ribose. Adenosine or adenosine derivatives play many important biological roles in addition to being components of DNA and RNA. For instance, adenosine is involved in cellular energy transfer and performs important signaling functions in both the peripheral and central nervous system [1,2]. It is well known that adenosine is a product of ATP degradation and its release from cells is a possible sign of disease. Therefore, the direct detection and monitoring of adenosine under physiological conditions have gained increasing significance in analytical, environmental, and biomedical applications.

Several traditional assays, such as high-performance liquid chromatography, UV-absorbance, Raman spectroscopy and fluorescence spectroscopy, have been applied for the detection of adenosine [3,4,5,6,7,8]. These techniques present the main problems of high detection limit and low selectivity. With the development of biotechnology and nanotechnology, enzymes and aptamers with specific molecular recognition ability have been used to fabricate different biosensors for the highly sensitive and selective detection of adenosine [9,10,11,12,13,14]. For example, Zhang *et al.* reported an electrochemical biosensor for detecting adenosine based on a structure-switching aptamer and the subsequent amplification with DNA-modified nanoparticles [10]. Li and co-workers demonstrated an aptamer biosensor based on surface-enhanced Raman scattering, and obtained a detection limit of 12.4 pM [11]. However, these biosensors suffer from drawbacks due to the complicated synthesis of DNA-modified nanoparticles and the labeling of probes and targets. Therefore, developing simpler, label-free adenosine biosensors with high sensitivity and selectivity is desired.

Atomic force microscopy (AFM)-based single-molecule force spectroscopy (SMFS) allows for the measurements of tiny forces associated with formation and breaking of single hydrogen bonds. It has therefore been widely used to study the specific molecular recognition interactions in antigen-antibody [15], ligand-receptor [16,17], and complementary ssDNA [18] pairs. SMFS is also powerful for studying any function and property of biomolecules associated with force changes, and especially for measuring the adsorption force between biomolecules and functional nanomaterials [19,20,21,22]. AFM-based SMFS can also be employed as a promising label-free biosensing technique with high sensitivity. Until now, there are a few reports on the detection of biomolecules with SMFS [23,24,25,26]. For example, Zhang and co-workers reported SMFS-based detection of DNA mismatched hybridization [23]. Nguyen *et al.* reported the detection of adenosine monophosphate, with a detection limit of 3.7 ± 2.5 µM [24]. Recently, we presented an SMFS-based, label-free bioanalytical system capable of selectively sensing the presence of specific ssDNA oligomers and proteins with sub-nm sensitivity [25].

In this work, we would like to explore the potential of AFM-based SMFS for the label-free detection of adenosine. To achieve this aim, an adenosine aptamer was bound onto the AFM tip, and the corresponding force-distance (FD) curves between the aptamer and a graphite surface were measured by SMFS until complete detachment, providing a reference desorption force. After that, low-concentrated adenosine was added into the liquid cell to bind to the aptamer. The formation of an adenosine-aptamer complex triggers a DNA conformational transition, which is associated with a change of the FD curve and in particular of the desorption force from graphite. Based on the obtained experiments, we have proven that our SMFS-based biosensor can be utilized to effectively detect adenosine in the range of 0.1 to 1 nM. In addition, our biosensor presents a very high selectivity for adenosine against uridine, guanosine, and cytidine. Our strategy is very simple but powerful, being mainly based on molecule-molecule and molecule-material recognitions. We expect that similar SMFS-based sensing strategies will be developed in the near future to detect a wide range of other analytes at sub-nM concentrations.

## 2. Experimental Section

### 2.1. Materials and Reagents

A highly oriented pyrolytic graphite (HOPG) wafer with ZYB quality (10 × 10 mm^2^) was purchased from NT-MDT (Moscow, Russia). Non-conductive silicon nitride AFM probes (DNP-S10) with a 45 ± 10 nm thick Ti/Au layer coated on the back side were obtained from Bruker Corporation (Palaiseau, France). The adenosine DNA aptamer (5*'*-NH_2_-(CH_2_)_6_-AGAGAACCTGGGGGAGTATTGCGGAGGAAGGT-3*'*) was synthesized by IBA (Göttingen, Germany), aliquoted, and stored at −20 °C. All other chemicals were bought from Sigma-Aldrich (Germany). The water used in this work is ultrapure water after purification with a Mill-Q Integral system (18.0 MΩ). The binding buffer is composed of 20 mM Tris-HCl (pH 7.4), 300 mM NaCl, and 5 mM MgCl_2_.

### 2.2. Preparation and Characterization of Flat Graphite Surfaces

Flat graphite surfaces were prepared by mechanically exfoliation of an HOPG wafer with Scotch^®^ tape [27]. The topography images of newly cleaved HOPG surface were examined by means of a NanoScience atomic force microscope (JPK Instruments AG, Berlin, Germany) using a bare tip in AC (tapping) mode. Commercially available non-conductive silicon nitride (Si_3_N_4_) AFM tips (DNP-S10) from Bruker Corporation (France) with a nominal spring constant of 0.35 N·m^−1^ were used. Topographical and deflection images were collected as 512 × 512 pixels with a lateral scan speed of 2.0 Hz.

### 2.3. Modification of AFM Probes

**Figure 1:**
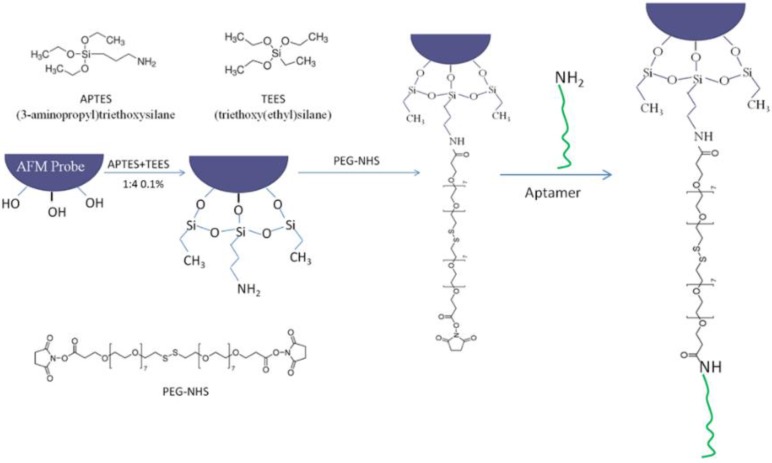
Modification of the AFM probes and conjugation of the ssDNA aptamer.

Figure 1 shows the procedure used for the modification of AFM probes. Before modification, all AFM probes were immersed in newly prepared Piranha solution (7/3 v/v, 98% H_2_SO_4_, 30% H_2_O_2_) for 30 min to remove possible organic contaminates. *Caution: the piranha solution has a very strong oxidizing power and is extremely dangerous to handle. Goggles, face shields, and gloves are needed for protection.* Then the probes were rinsed with large amount of ultrapure water and ethanol (99%) several times. The probes were then silanized by a mixed solution of 3-aminopropyl triethoxysilane (APTES) and triethoxy(ethyl)silane (TEES) (1% in toluene, 1/4 v/v, APTES/TEES) to functionalize their surfaces with amino groups. In this step, instead of immersing the whole probes into the mixed solution, they were hung vertically by tweezers over the solution and adjusted to submerge into the solution only a small part of the probe. This technique effectively reduces the unwanted functionalization of parts of the probes other than the tip, hence reducing the total amount of DNA aptamer linked to the probe and eventually reducing the adenosine detection limit. After 20 min immersion, the probes were rinsed with ethanol and ultrapure water. They were then transferred into 4,7,10,13,16,19,22,25,32,35,38,41,44,47,50,53-Hexadecaoxa-28,29-dithiahexapentacontanedioic acid di-N-succinimidyl ester (PEG-NHS ester disulfide (*n* = 7)) (0.1 mg/mL, 100 μL) for 1 h to bind the PEG-NHS ester disulfide to the AFM probes via covalent interaction between surface-bound NH_2_ groups and the NHS ester groups. The probes were subsequently rinsed with ultrapure water and immersed into the ssDNA aptamer solution (100 nM, 100 μL) for 30 min to link the aptamer to the probes. Finally, the aptamer-modified AFM probes were rinsed with a large amount of ultrapure water to remove non-covalently adsorbed DNA molecules prior to the SMFS experiments.

### 2.4. AFM-Based Force Spectroscopy Experiments

The force measurements were carried out with a NanoScience atomic force microscope (JPK Instruments AG, Berlin, Germany) in liquid cells. Cantilevers with a spring constant ranging from 0.32 to 0.68 N/m (according to measurement of their thermal fluctuations) were used. Graphite surfaces were freshly cleaved with Scotch^®^ tape prior to each experiment and immediately placed in a liquid cell, which was then filled with the binding buffer or other analyte solutions (vide infra). The spring constant of each AFM cantilever was calibrated in buffer by the thermal fluctuations method. During the SMFS experiments, the AFM probe was brought into contact with graphite, settled at the surface for 1 s and then pulled away, while the deflection of the cantilever and its displacement were measured at different locations on the graphite surface. For the detection of adenosine and the selectivity test against uridine, guanosine and cytidine, after filling the liquid cell with the analyte solution, the probe was kept submerged in the cell at a few hundred μm over the graphite surface for 1 h before starting the SMFS measurement. Typical parameters for the force measurements were: (1) Z-length: 0.4 μm; (2) moving speed of AFM probe: 0.5 μm·s^−1^; (3) extend time: 0.8 s; (4) delay time on substrate: 1 s. The 1 s delay ensures equilibrium interaction between the aptamer tethered to the AFM probe and the graphite surface.

### 2.5. Detection Limit and Selectivity Tests

The detection limit was tested by performing a series of SMFS experiments in liquid cells filled with adenosine binding buffer solutions at different concentrations (1 pM, 100 pM, 1 nM, 10 nM, 100 nM, and 1 μM).

The selectivity of our SMFS-based sensor architecture towards adenosine was verified by performing SMFS successive experiments in uridine, guanosine, cytidine and adenosine solutions (1 μM in binding buffer). All the experiments were conducted under the same conditions.

### 2.6. Effects of Loading Rates and Solutions

We define here the loading rate *r* as *r* = d*f*/d*t* = *vk*_eff_, where *v* is the pulling velocity, and *k*_eff_ is the effective spring constant of the AFM probe. It is therefore not the local loading rate acting on the surface/DNA bonds (which depends on the stiffness of the DNA linker and its precise adsorption configuration on graphite) but the nominal loading rate of the AFM cantilever. To test the effect of the loading rate on the desorption force in our experiments, we carried out a series of SMFS measurements both in pure binding buffer and in the presence of adenosine (1 μM) with different pulling velocities ranging from 0.1 to 2 μm·s^−1^.

To test the effect of different media on the desorption force between the DNA aptamer and the graphite surface, another set of SMFS experiments was performed using, instead of the standard binding buffer, also ultrapure water; 50 mM NaCl solution; PBS buffer (10 mM phosphate buffer, 2.7 mM KCl and 137 mM NaCl, pH 7.4), both without and with adenosine (1 μM).

### 2.7. Statistic Analysis

All the data were analyzed with the JPK SPM Data processing program (Version 4.3.11). For the statistical analysis, all data were expressed as means ± standard deviation (SD) for *n* > 50 (*n* represents the number of data being analyzed). The statistical analysis was conducted with the software Origin 8 (version 8.0724; OriginLab Corp., Northampton, USA, 2007) at a confidence level of 95%.

## 3. Results and Discussion

### 3.1. Sensing Principle

**Figure 2:**
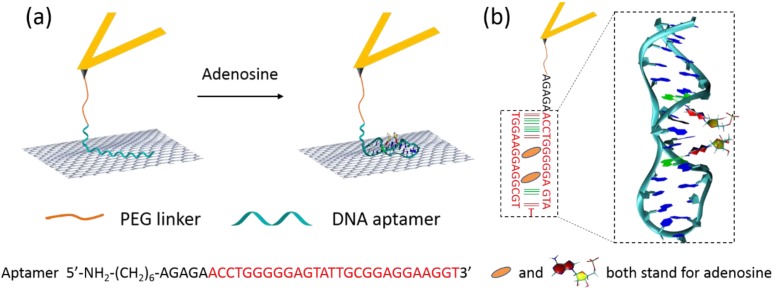
(**a**) Schematic representation of our AFM-based SMFS detection of adenosine. (**b**) Scheme and 3D structure of the adenosine/aptamer complex (PDB ID: 1AW4) [28].

Figure 2 demonstrates the construction and detection principle of our SMFS-based sensing architecture. A 32-m ssDNA aptamer with the sequence showed in the picture (Figure 2a) was tethered onto the AFM probe through a PEG-NHS linker. The effective adenosine-binding motif is highlighted in red. The probe was then brought into contact with a freshly cleaved HOPG surface. In the absence of adenosine, the ssDNA molecule is expected to lay flat on the graphite surface as a random-coil and to interact with the graphite surface through π-π stacking interactions [19,20,21]. Upon retraction of the AFM probe, the adsorbed bases of the ssDNA can slide freely on the flat graphite surface, resulting in a steady-state peeling force [19,20].

In the presence of adenosine, the aptamer will undergo a conformational transition after formation of a DNA/adenosine complex. Figure 2b shows the schematic structure of the aptamer after binding with adenosine. A 3D structure of the binding motif is also reported, based on its determined atomistic structure (PDB-ID: 1AW4) [28]. The strength of the interaction between the adenosine-bound, folded aptamer and the graphite surface will differ from that without adenosine. Therefore, different FD curves and desorption forces will be recorded in SMFS experiments in the absence and presence of adenosine, allowing for detection of adenosine above a certain concentration. The detection limit is set by both the binding kinetics, or, under equilibrium conditions, by the binding affinity of adenosine for its aptamer.

### 3.2. Typical FD Curves before and After Adding Adenosine

**Figure 3:**
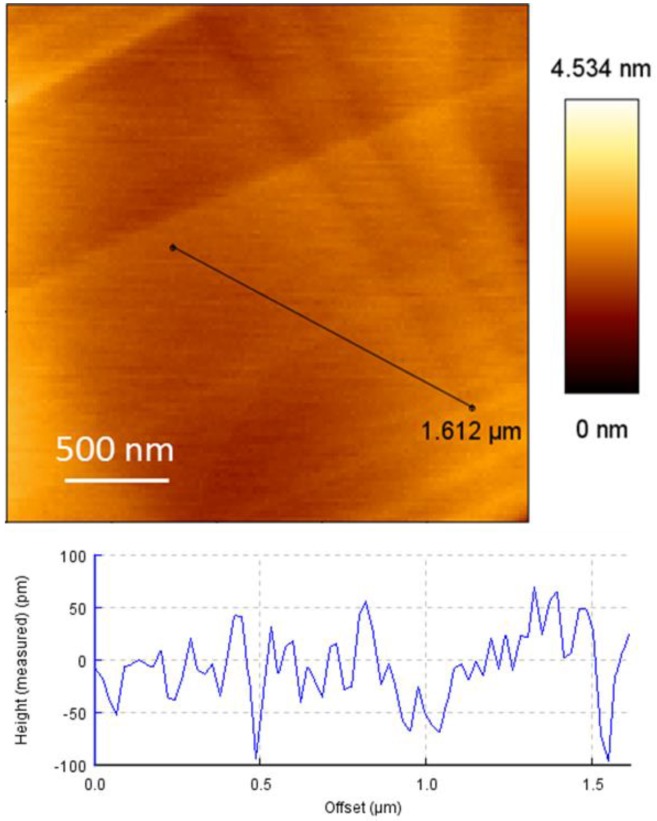
Typical AFM height image and corresponding cross-section analysis of the graphite surface prepared by mechanical exfoliation of an HOPG wafer.

Flat graphite surfaces were prepared by mechanical exfoliation of an HOPG wafer with Scotch^®^ tape [25]. Before the force spectroscopy measurements, the flatness of graphite surface was first checked by randomly scanning five different areas (2.5 × 2.5 μm) using a bare AFM probe in AC mode in air. Figure 3 shows a typical AFM height image. From the cross-section analysis, a mean roughness of about 0.21 ± 0.07 nm in the 2.5 μm × 2.5 μm area was found, which is definitely flat enough to perform the SMFS experiments.

**Figure 4:**
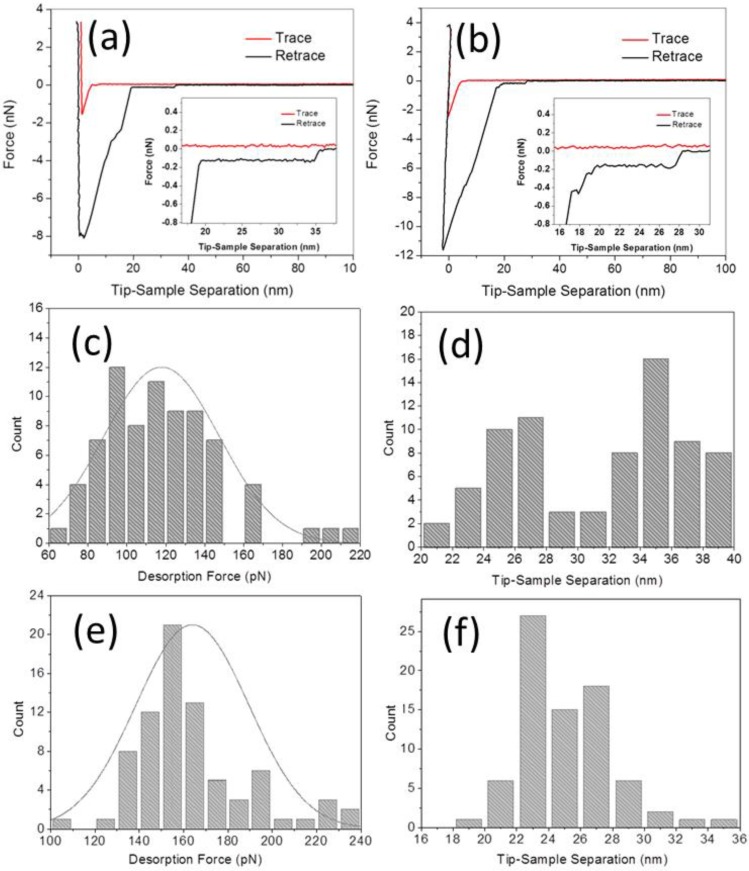
SMFS experiments and statistical analysis: (**a**,**b**) Typical FD curve by peeling aptamer from graphite surface (**a**) before and (**b**) after adding 1 μM adenosine; (**c**,**d**) Distributions of the desorption forces and tip-sample separations corresponding to the FD curves of (**a**); (**e**,**f**) same, for the FD curves of (**b**). After the adding of adenosine, the mean desorption force by peeling aptamer from graphite surface increased from 117.8 ± 29.5 to 164.3 ± 25.4 pN, and the mean tip-sample separation decreased from about 36.1 ± 2.0 to 25.2 ± 3.4 nm.

SMFS measurements were then performed between the aptamer-tethered AFM probe and the graphite surface in binding buffer and adenosine solution (1 μM in binding buffer), respectively. Before the SMFS measurement in the presence of adenosine, the AFM probe was kept submerged in the solution (a few hundred μm above the graphite surface) for 1 h to allow enough time for the molecular recognition between aptamer and adenosine to take place. Typical FD curves obtained for both systems under a loading rate of 3.29 × 10^5^ pN·s^−1^ are presented in Figure 4a,b, respectively. The approaching traces of both FD curves display a small jump-to-contact force at about 5 nm separation, probably due to the initial contact between the hanging ssDNA strand and the graphite surface [19]. On retraction, an initial large adhesive force is observed in both FD curves, which is associated to the nonspecific adhesive junction between the monolayer coating of the AFM probe and the hydrophobic surface [19,20,21]. After the initial pull-off, there is a large drop in force, but a roughly constant force persists with increasing tip-sample separation in both curves. This stable force plateau is interpreted as the progressive desorption of a single ssDNA strand from the graphite surface [19,22,25]. Importantly, for long enough PEG linkers and DNA aptamers, the non-specific interaction between probe and surface will not affect the value of the plateau force. In the absence of adenosine, in many cases the length of the plateau is up to the contour length of the fully extended 32-m ssDNA (approximately 18 nm). In some of the curves, the force decreases in characteristic discrete steps (not shown here), which we interpret as resulting from the successive detachment of a small number of ssDNA strands.

To analyze the force data, for each FD curve we measure the magnitude of the plateau steps as the difference between the average force value estimated over a distance of 1 nm just before and after the force jump. Figure 4c shows the distribution of the plateau force values before adding adenosine, which is characterized by a mean desorption force of 117.8 ± 29.5 pN (±SD, *n* = 75). This value increased to 164.3 ± 25.4 pN (*n* = 79) after adding 1 μM adenosine (Figure 4e). This ~40% increase of the peeling force is obviously associated to the conformational transition of the aptamer after molecular recognition with adenosine.

Besides the force enhancement, also a significant reduction of the tip-sample separation is observed after the addition of adenosine. Namely, in the absence of adenosine the distribution of tip-sample separation presents a first peak centered at about 26 nm and a second, slightly larger peak between 32 and 40 nm with a mean value is 36.1 ± 2.0 nm (Figure 4d). The latter corresponds to a plateau length of 14 to 18 nm (*i.e.*, close to the contour length of the ssDNA aptamer). In the presence of adenosine, instead, the distribution of tip-sample separations clusters between 22 and 30 nm, with a mean value of 25.2 ± 3.4 nm (mostly corresponding to a plateau length of less than 12 nm in the obtained FD curves), as shown in Figure 4f. The decrease in tip-sample separation confirms the successful binding of the ssDNA aptamer with adenosine molecules, which leads to the formation of a folded, hairpin-like DNA structure [2,14,29] and thus to a statistically reduced length of the adsorbed strand.

The interaction force of the complex with graphite surfaces has never been studied before. For the first time, our SMFS finding indicates that the hairpin-folded DNA structure stabilized by adenosine has a stronger interaction with graphite than the pure ssDNA strand. The behavior of the folded aptamer/adenosine complex is thus different from the one of a fully hybridized dsDNA helix structure, which shows a smaller adsorption force on graphite compared to each individual ssDNA strand [25]. We speculate that the intercalated adenosine molecules within the adsorbed strand hinders a smooth base-after-base detachment upon AFM pulling, effectively increasing the cooperativity of binding of several bases to the surface and therefore leading to a statistically increased adhesion force. Notably, the folded structure does not present perfectly matched double-helical folding, which is a strict prerequisite for a decrease of interaction force to graphite. In fact, we have previously observed that even a single base-pair mismatch in a dsDNA oligonucleotide leads to higher adhesion forces, comparable to the ones of ssDNA.

### 3.3. Detection Limit Test

To evaluate the sensitivity of our sensor architecture, different concentrations of adenosine from one stock solution were added into the liquid cell before performing the SMFS measurements.

**Figure 5:**
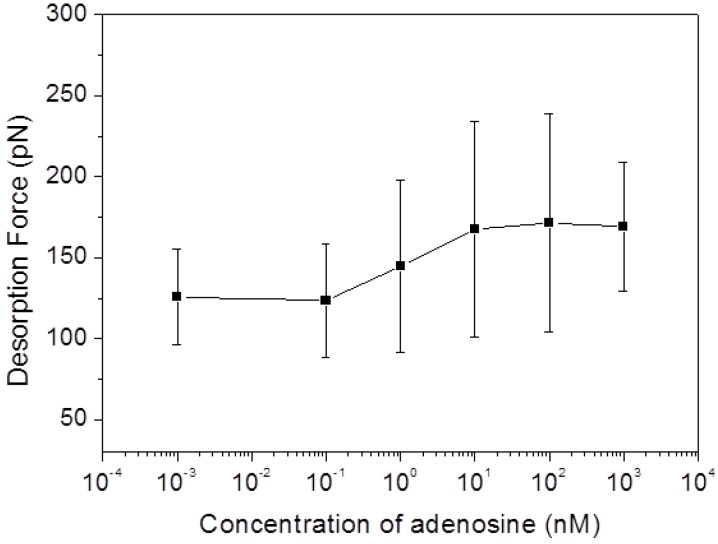
Mean desorption forces from SMFS experiments conducted in binding buffer with different concentrations of adenosine (1 pM, 100 pM, 1 nM, 10 nM, 100 nM, and 1 μM).

Figure 5 shows the SMFS force responses to different concentration of adenosine. It can be seen that at very low adenosine concentration (less than 100 pM) the mean desorption force is around 125 pN (for example, the force is 125.9 ± 29.5 pN in 1 pM adenosine, and 123.3 ± 35.1 pN in 100 pM adenosine), which remains at the same level as the desorption force obtained in binding buffer without adenosine (123.9 ± 18.0 pN). After increasing of the adenosine concentration to 1 nM, a first force increase (144.7 ± 52.8 pN) is observed. The further increase of the adenosine concentration to 10 nM caused a further enhancement of the detachment force to 167.5 ± 66.5 pN, after which it remains constant with adenosine concentration at a level around 170 pN. These results confirm the enhancement of the adhesion force between the folded aptamer/adenosine complex and the graphite surface and suggest a detection limit of our sensor architecture in the range of 0.1 to 1 nM.

### 3.4. Selectivity of the Fabricated Sensor Architecture

**Figure 6:**
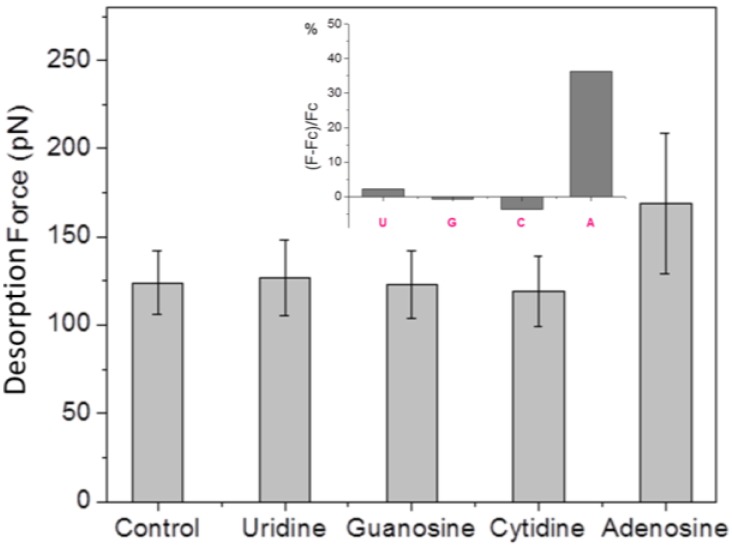
Mean desorption force obtained in SMFS measurements performed in pure binding buffer (control), and in the presence of uridine (U), guanosine (G), cytidine (C), or adenosine (A). The inset shows the relative force increase after addition of each analyte to the binding buffer.

To investigate the selectivity of our sensor architecture towards adenosine, we performed additional SMFS measurements after adding to the binding buffer uridine, guanosine, cytidine or adenosine at the same concentration (1 μM). As before, an SMFS experiment in binding buffer alone was performed as control. All the experiments were conducted under the same conditions. Each time after adding the analyte (uridine/guanosine/cytidine/adenosine) to the liquid cell, the AFM probe was kept away from the surface in the bulk solution for 1 h before force measurement, to allow for aptamer/analyte molecular recognition. The obtained mean desorption forces in the four cases are shown in Figure 6. It can be clearly seen that addition of uridine, guanosine or cytidine does not lead to a change of the desorption force with respect to pure buffer, whereas the addition of adenosine again caused the adsorption force to increase. The relative force increase (F − F_c_)/F_c_ (here F is the mean desorption force in the presence of analyte and F_c_ is the control desorption force in pure buffer) is about 36% for the case of adenosine, and negligible for the other nucleosides, as shown in the inset of Figure 6.

### 3.5. Factors Affecting the Test

**Figure 7:**
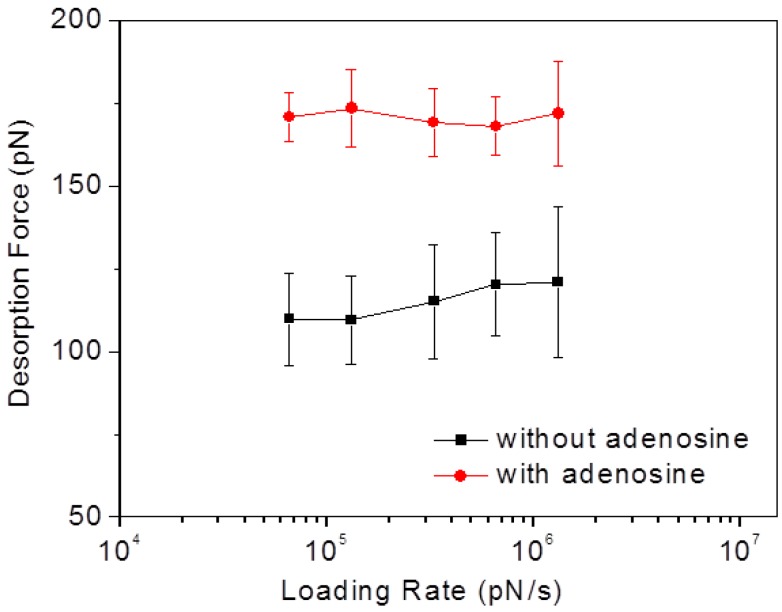
Mean aptamer/graphite desorption forces plotted logarithmically against the corresponding loading rates in binding buffer without and with adenosine.

To test the effect of loading rate on the desorption force of our system, we conducted a series of SMFS measurements both in binding buffer alone and in adenosine solution (1 μΜ in binding buffer) with different AFM probe retracting velocities ranging from 0.1 to 2 μm·s^−1^, corresponding to a loading rate range of 6.6 × 10^4^ to 1.3 × 10^6^ pN·s^−1^ (here the spring constant of AFM probe was determined to be 0.66 N/m). The obtained mean desorption forces are shown in Figure 7. Within the test range, no obvious trend in the variation of the desorption force can be observed in either case, considering the large error bars associated with the measurement. This result is agreement with the previous report by Vezenov and co-workers [19], who suggested that the loading rate has no significant effect on the desorption force of ssDNA from graphite, for sufficiently small retracting velocities.

**Figure 8:**
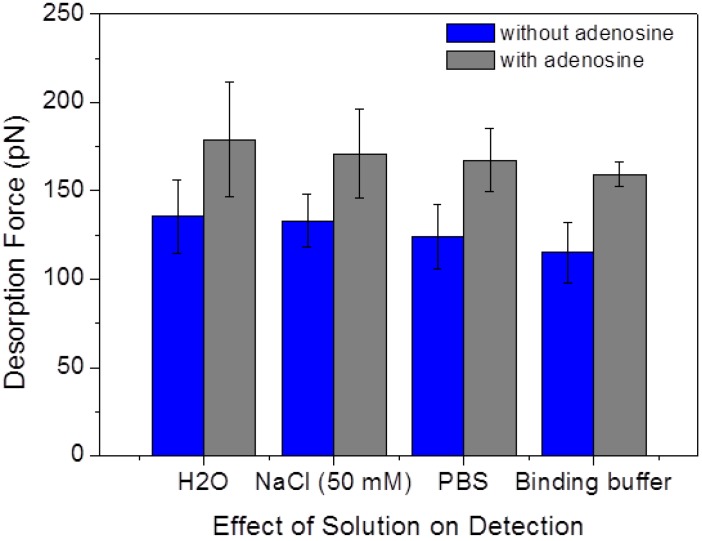
Mean aptamer/graphite desorption forces without and with adenosine (1 μM) in four different water solutions.

To test the effect of different media solutions on the aptamer/graphite adhesion force, SMFS measurements in the absence and presence of 1 μM adenosine were performed in four different solutions; namely in ultrapure water, 50 mM NaCl solution, PBS buffer (with 137 mM NaCl), and in the binding buffer used in all the other experiments. The resulting desorption forces are depicted in Figure 8. It can be seen that the presence of adenosine results in a clear force increase in all solutions, suggesting that the desorption mechanisms are widely independent of the precise composition of the water environment. It should be noted that the FD curves obtained in the binding buffer presents the least variability (both in terms of the number of curves showing successful surface/aptamer binding and in terms of similar plateau lengths and heights), as indicated by the smaller width of the force distribution (error bars in Figure 8) with respect to the other solutions.

In addition, the mean desorption force show a slight decreasing trend in the order of ultrapure water, NaCl solution, PBS buffer, and binding buffer, both without and with adenosine. As the ionic strength in these solution increases in the corresponding order, this slight decrease in the desorption force is consistent with our previous finding that the interaction strength between ssDNA and graphite decreases with increasing NaCl concentration [22]. In fact, DNA/graphite interactions are mostly due to van der Waals forces and hydrophobic effect (including π-stacking interactions) [19,30], and are thus only indirectly influenced by dissolved ions. These cause a small decrease of the end-to-end distance of ssDNA strands, partially shielding the hydrophobic surface/molecule interactions [22].

## 4. Conclusions

In summary, we have demonstrated a novel SMFS-based sensor architecture for the highly sensitive and selective detection of adenosine. This sensor takes advantage of the specific molecular recognition between adenosine and an appropriate DNA aptamer, as well as of the intrinsic SMFS sensitivity. We have reached a relatively low adenosine detection limit in the range of 0.1–1 nM and very good selectivity against uridine, guanosine and cytidine. The loading rate and the precise composition of the aqueous solution show negligible effects on the adenosine detection capability. Interestingly, we could show that the folded, hairpin-like structure of the adenosine/aptamer complex has a stronger interaction with graphite than the ssDNA aptamer alone. This simple but powerful SMFS-based biosensing technique is very promising for the detection of a wide range of other analytes, since aptamers for numerous molecules have been reported and the methods for discovering new aptamers are also well-established.
